# Enlarged accessory spleen after splenectomy mimicking a pancreas tumor

**DOI:** 10.1016/j.ijscr.2020.12.037

**Published:** 2020-12-16

**Authors:** Le Tuan Linh, Thieu-Thi Tra My, Bui Van Lenh, Tran-Van Giang, Luong Viet Bang, Nguyen Minh Duc

**Affiliations:** a Department of Radiology, Ha Noi Medical University Hospital, Ha Noi, Viet Nam; b Department of Radiology, Ha Noi Medical University, Ha Noi, Viet Nam; c Department of Pathology, Ha Noi Medical University, Ha Noi, Viet Nam; d Department of Radiology, Pham Ngoc Thach University of Medicine, Ho Chi Minh City, Viet Nam; e Department of Radiology, Children’s Hospital 2, Ho Chi Minh City, Viet Nam

**Keywords:** Accessory spleen, Enlargement, Splenectomy, Pancreatic tumor

## Abstract

**Introduction:**

An accessory spleen (AS), a common condition, is usually located in the hilar region of the spleen. ASs are not often large; however, after splenectomy, the initially inactive AS may become reactive and hypertrophic. Therefore, an AS can be misdiagnosed as a neoplastic tumor and removed unnecessarily. An undiagnosed abdominal mass located in the spleen site in a patient who has had a splenectomy must be managed carefully. Computed tomography (CT) scanning and magnetic resonance imaging (MRI) may provide useful information for the diagnosis, preventing unnecessary surgery.

**Case presentation:**

Herein, we report the case of a 38-year-old female with an enlargement of AS after splenectomy that was misdiagnosed as a primary tumor of the pancreas and managed by a nonessential surgery.

**Conclusion:**

An AS should be added to the differential diagnosis of a pancreatic tail tumor for patients with prior splenectomy in order to avoid nonessential surgery to ensure the patient’s safety.

## Highlights

• Enlarged accessory spleens are often misdiagnosed as neoplasms.
• Imaging features and histopathology of accessory and normal spleens are similar.
• Accessory spleen should be considered in differential diagnosis of abdominal tumors.

## 1 Introduction

An accessory spleen (AS), also known as a supernumerary spleen, is a small nodule of healthy splenic tissues that are separated from the main body of the spleen [1]. ASs are relatively common and are seen in 10%–30% of the population. The splenic hilus, followed by the pancreatic tail, are the two most common sites of an AS [2]. Patients with ASs are usually asymptomatic, and ASs are typically discovered incidentally in radiological examinations. In patients who have had a splenectomy, ASs become enlarged [1]. ASs often appear as well-circumscribed, round masses that are supplied by small branches of the splenic artery [3,4]. An AS usually does not require treatment; thus, it is crucial to make an accurate diagnosis preoperatively. In this article, we aimed to illustrate a case of an enlarged of AS after splenectomy that was misdiagnosed as a primary pancreatic tumor for which the patient was subjected to an unnecessary operation.

## 2 Case description

**Fig. 1:**
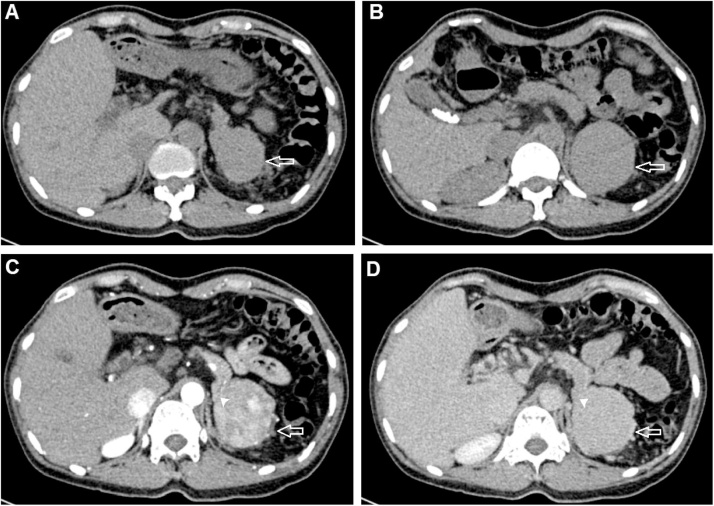
Axial CT scanner precontrast (A and B), in the arterial phase (C), and in the venous phase (D). There was a mass located in normal spleen site exhibiting isodensity with hepatic tissues (A and B, arrow), inhomogeneous enhancement in the arterial phase (C, arrow), and homogeneous enhancement in the venous phase (D, arrow). It was suspected that the mass developed from the pancreas tail (C and D, arrowhead).

**Fig. 2:**
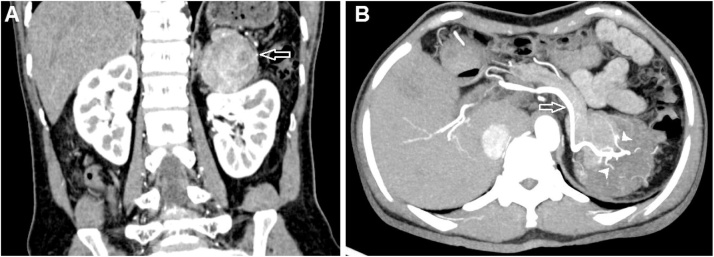
Coronal reconstruction of CT scan in the arterial phase (A) and axial maximum intensity projection in the arterial phase (B). The mass had well-defined borders and compressed the left kidney (A, arrow). The arteries feeding this mass (B, arrowhead) was a branch of the splenic artery (B, arrow).

**Fig. 3:**
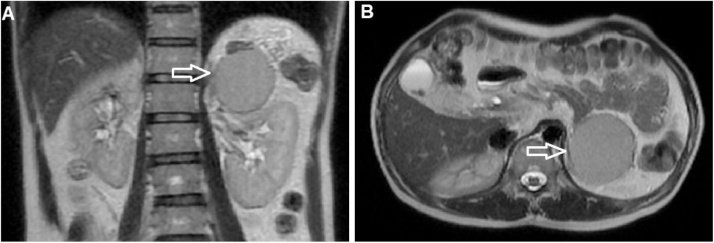
Coronal T2-weighted image (A) and axial T2-weighted image (B). Coronal T2-weighted image showed that the mass was located in the normal spleen site (arrow) and compressed the left kidney. The mass was well-circumscribed and had a higher signal than liver on axial T2-weighted image (B, arrow).

**Fig. 4:**
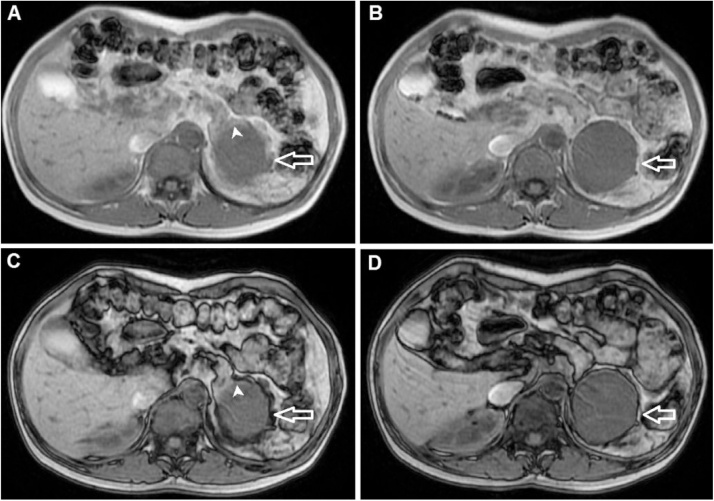
Axial in-phase T1-weighted images (A and B) and axial opposed-phase T1-weighted images (C and D). Axial T1-weighted images showed that the mass had low signal intensity compared to liver parenchyma (A and B, arrow) and did not lose signal intensity on T1 opposed-phase images (C and D, arrow). Part of the mass was suspected to be connected to the pancreas tail (A and C, arrowhead).

**Fig. 5:**
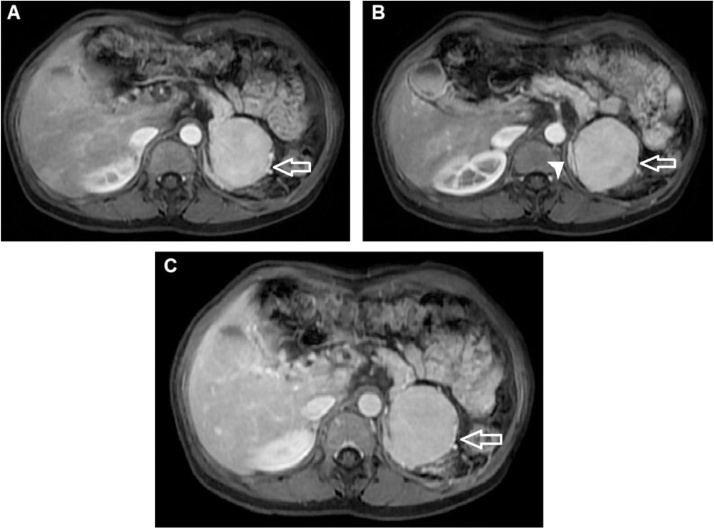
Axial T1-weighted images in the arterial phase revealed that the mass was markedly and inhomogeneously enhanced (A, arrow) and the left adrenal gland was normal (B, arrowhead). Axial T1-weighted images in the venous phase showed that the mass exhibited homogeneous enhancement (C, arrow).

**Fig. 6:**
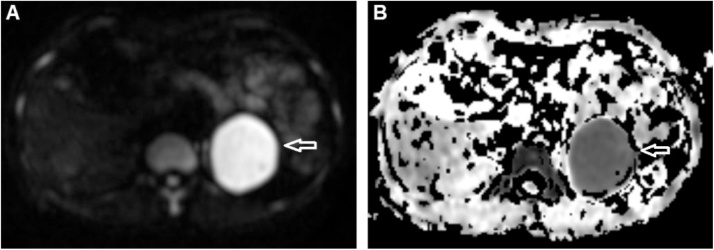
Axial diffusion-weighted images (A) and axial apparent diffusion coefficient (ADC) map (B). This mass had high signal intensity on diffusion-weighted image (A, arrow) and low signal intensity on ADC map (B, arrow) compared to liver parenchyma.

**Fig. 7:**
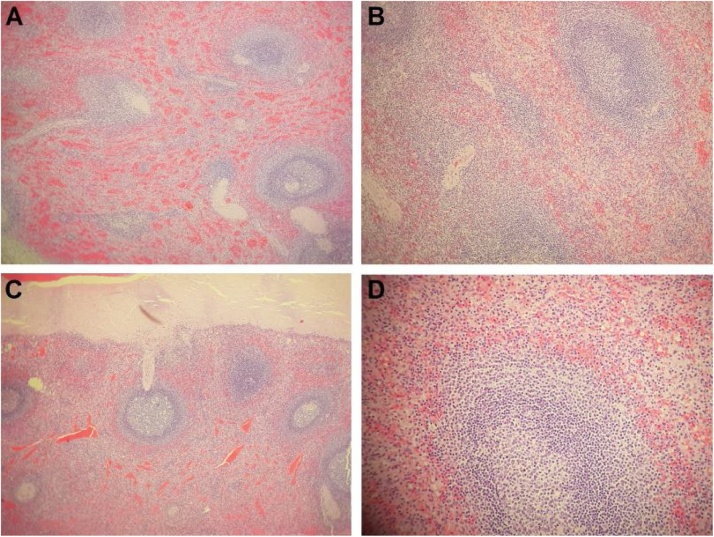
Microscopic appearance. (A) ×40: White pulp, red pulp, and connective tissue that presented within the mass as trabeculae that carry the arteries and veins. (B) ×100: The white pulps included periarterial lymphatic sheath, lymphatic follicles, and the marginal zone. (C) ×40: The mass with a fibrous capsule. (D) ×200: The lymphatic follicles included benign lymphocytes.

A 38-year-old female underwent a medical check-up at our institution. The patient’s medical profile had no history of weight loss, anorexia, or weakness. She had had a splenectomy 20 years prior for traumatic splenic rupture. Laboratory tests, including complete blood count, liver function test, and alpha-fetoprotein levels, were within the normal range. An abdominal CT scan without contrast enhancement revealed the absence of a normal spleen, with a homogeneous mass situated at the site (Fig. 1A and B). The mass exhibited heterogenous enhancement in the arterial phase (Fig. 1C), but homogeneous enhancement in the venous phase (Fig. 1D). The mass was suspected to have originated from the pancreas tail (Fig. 1A and C). There was no fat stranding surrounding the mass (Fig. 1). Coronal CT image revealed that this mass compressed the left renal (Fig. 2A). The mass was fed by several branches of the splenic artery (Fig. 2B). An abdominal MRI was performed. This confirmed that the mass was located in the normal site of spleen and compressed the left kidney (Fig. 3A) and that the mass was round, with well-defined borders and without fat stranding. The mass presented as hyperintense compared to liver parenchyma on T2-weighted image (Fig. 3B). T1-weighted images showed that the mass was hypointense compared with liver parenchyma and did not lose signal on T1 out-of-phase images (Fig. 4). Part of the mass was suspected to be connected to the pancreas tail (Fig. 4A and C). T1-weighted image in the arterial phase revealed that the mass enhanced markedly and inhomogeneously (Fig. 5A). The normal left adrenal gland was observed clearly (Fig. 5B). The mass exhibited homogeneous enhancement in the venous phase (Fig. 5C). It was clear that the mass showed restricted diffusion, since it was hyperintense on diffusion-weighted image (DWI) and had hypointense apparent diffusion coefficient (ADC) values on an ADC map compared to liver parenchyma (Fig. 6). The preoperative diagnosis was a tumor of the pancreas tail. The patient underwent an operation to remove the mass by a general surgeon with 10-year experience. Histopathology showed white and red pulps, as well as connective tissue that presented within the mass as trabeculae that carry the arteries and veins (Fig. 7A). The white pulps included periarterial lymphatic sheath, lymphatic follicles, and the marginal zone (Fig. 7B). It was surrounded by a capsule composed of dense fibrous tissue (Fig. 7C). The lymphatic follicles contained benign lymphocytes (Fig. 7D). Histopathology of the mass revealed the appearance of a spleen parenchyma. The final diagnosis was an enlargement of AS after splenectomy. This patient was discharged from the hospital 4 days after surgery without further complications or treatment. The patient was lost to follow-up later. This case was reported in line with the SCARE 2020 criteria [5].

## 3 Discussion

An AS is defined as ectopic splenic tissue that develops during embryonic growth [6]. ASs are commonly located in the hilum of the spleen (75% of cases) and near the tail of the pancreas (approximately 20% of cases); other sites are the stomach, the small and large intestine, and the pelvis [7]. Patients with AS are often asymptomatic, but may present with an abdominal mass or adverse events such as torsion, hemorrhage, spontaneous rupture, or cyst formation [8]. ASs receive their vascular supply from branches of the splenic artery [4]. They are usually solitary, but in some patients, there are two or more [7]. ASs contain normal spleen tissue; thus, they also have immunologic functions [8]. The size of an AS is typically approximately 1–2 cm in diameter [9]. However, in patients after splenectomy, the AS can be expanded and mimic a neoplasm such as a pancreas tumor, stomach submucosal tumor, or retroperitoneal tumor [1,10,11]. Because ASs are congenital foci of healthy splenic tissues, they have several imaging features similar to normal spleen [12]. ASs are generally round and well-circumscribed, and their enhancement is identical to that of the normal splenic parenchyma in both the arterial and venous phases after contrast injection [3]. In this case, the AS enhanced heterogeneously in the arterial phase and homogeneously in the venous and delayed phases. CT scans with contrast agent may show vascular branches from the splenic artery supplying the ASs. On MRI, the ASs had the same signal intensity features as normal spleens in all sequences [12]. They are hypointense on T1-weighted and hyperintense on T2-weighted images. On DWIs, ASs are hyperintense, while they are hypointense on ADC map because they are rich in lymphocytes [13]. They exhibited heterogeneous enhancement immediately in the arterial phase, becoming homogeneous in the venous phase [14]. However, a small AS would exhibit homogeneous enhancement in the arterial phase [3]. Microscopically, ASs are described as being similar to normal spleens. An AS typically has a well-defined fibrotic capsule [15]. It contains white and red pulp. The white pulp consisted of a periarteriolar lymphocyte sheath, lymphoid follicles with germinal centers, and a mantle region surrounded by a loosely distributed marginal zone [16]. The red pulp was composed of a three-dimensional meshwork of splenic cords and venous sinuses [16]. The combination of medical history, CT, and MRI provide useful information for diagnosis. However, sometimes it is necessary to perform a biopsy to determine the origin of the lesion [17]. With medical history and typical imaging, the diagnosis could be confirmed without surgery, allowing patients to avoid surgical complications and removal of splenic tissue.

In this case report, the patient had had a splenectomy many years prior. As the lesion was related to the tail of the pancreas, and due to lack of experience, the patient received an unnecessary surgery. The mistake was thought to be an exclusion of an AS in the preliminary diagnosis for a patient with prior splenectomy.

## 4 Conclusion

AS is a common congenital condition typically without severe symptoms. CT and MRI features of an AS are the same as those of the normal spleen. In patients who have had a splenectomy, ASs may be enlarged and can mimic a tumor. Clinician should take into consideration that for patients who have had a splenectomy, an AS must be included in the differential diagnosis of a pancreatic tail tumor in order to avoid nonessential surgery like that described in the present report.

## Declaration of competing interest

The authors report no declarations of interest

## Funding

This research did not receive any specific grant from funding agencies in order to execute this case report.

## Ethical approval

The study is exempt from ethical approval.

## Consent

Informed consent of patient was obtained.

## Author’s contribution

LTL and NMD contributed equally to this article as co-first authors. Conceptualization: LTL and NMD; data curation: Thieu-Thi TM and NMD; formal analysis: LTL and NMD; writing—original draft: Thieu-Thi TM and NMD; writing—review and editing: LTL, Thieu-Thi TM, and NMD; All authors have read and agreed to the published version of the manuscript.

## Registration of research studies

Not applicable.

## Guarantor

The Guarantor’s of this article was Nguyen Minh Duc.

## Provenance and peer review

Not commissioned, externally peer-reviewed.

## Data availability statement

Data sharing is not applicable to this article as no datasets were generated or analyzed during the current study.
